# Concurrent irinotecan, oxaliplatin and UFT in first-line treatment of metastatic colorectal cancer: a Phase I study

**DOI:** 10.1038/sj.bjc.6603521

**Published:** 2007-01-09

**Authors:** H Y Sheikh, J W Valle, K Palmer, A Sjursen, O Craven, G Wilson, R Swindell, M P Saunders

**Affiliations:** 1 Department of Clinical Oncology, Christie Hospital NHS Trust, Manchester M20 4BX, UK; 2 Department of Medical Oncology, Christie Hospital NHS Trust, Manchester M20 4BX, UK; 3 Department of Medical Statistics, Christie Hospital NHS Trust, Manchester M20 4BX, UK

**Keywords:** advanced colorectal cancer, CPT-11, irinotecan, oxaliplatin, tegafur–uracil, UFT

## Abstract

The feasibility of combining UFT plus leucovorin (LV) with alternating irinotecan and oxaliplatin was investigated in the first-line treatment of patients with advanced colorectal cancer. Twenty-five patients, median age 63 (range 24–79) years, World Health Organisation performance status 0–2 and median four marker lesions, received irinotecan 180 mg m^−2^ on day 1, oxaliplatin 85–100 mg m^−2^ on day 15 and UFT 200–300 mg m^−2^ day^−1^ with LV 90 mg day^−1^, days 1–21 of a 28-day cycle. Patients were treated in cohorts of three. At the highest dose (irinotecan 180 mg m^−2^, oxaliplatin 100 mg m^−2^ and UFT 300 mg m^−2^ day^−1^), three of four patients experienced grade 3 toxicity. Diarrhoea, lethargy and vomiting were dose-limiting. Three of nine patients had grade 2 toxicities at the maximum tolerated dose (irinotecan 180 mg m^−2^, oxaliplatin 100 mg m^−2^ and UFT 250 mg m^−2^ day^−1^). There were no grade 3 toxicities in the first month of therapy. The overall response rate was 71% in 21 evaluable patients; progression-free survival was 8.8 months. Alternating irinotecan and oxaliplatin plus UFT is an effective and well-tolerated first-line treatment for patients with advanced colorectal cancer. We recommend a dose of irinotecan 180 mg m^−2^ on day 1, oxaliplatin 100 mg m^−2^ on day 15 and UFT 250 mg m^−2^ day^−1^ with LV 90 mg day^−1^ on days 1–21 of a 28-day cycle for future studies.

The lifetime risk of developing colorectal cancer is one in 20 in the UK (Office for National Statistics, 2004). Around 31 000 cases are diagnosed annually and over 18 000 patients die as a result of the disease, making colorectal cancer the second most common cause of cancer death. For patients with unresectable local or metastatic disease, either at the time of presentation or at relapse (collectively termed ‘advanced disease’), the prognosis is poor. Since the advent of 5-fluorouracil (5-FU) in the treatment of colorectal cancer nearly 50 years ago, subsequent refinement of administration regimens for use in such patients have only improved their median survival from 6 to 9 months to approximately 12 months (Quinn *et al*, 2001). The aim of treating these patients is, therefore, to improve both the duration and quality of their remaining life.

5-Fluorouracil is now used in combination with newer drugs that have nonoverlapping mechanisms of action and hence no crossresistance with 5-FU. One such agent is irinotecan, a topoisomerase I inhibitor that has been shown to improve response rates, increase median times to progression and treatment failure, and provide significant survival benefits when used in combination with 5-FU/leucovorin (LV) compared with 5-FU/LV alone (Douillard *et al*, 2000; Saltz *et al*, 2000). Oxaliplatin (Wiseman *et al*, 1999) is also active in colorectal cancer and response rates of 40–50% have been demonstrated for the combination of oxaliplatin and 5-FU/FA (de Gramont *et al*, 2000; Giacchetti *et al*, 2000).

The oral fluoropyrimidines UFT (tegafur:uracil in a 4 : 1 ratio) and capecitabine are more convenient and acceptable forms of chemotherapy than intravenous 5-FU and have been shown to have equivalent efficacy to intravenous bolus 5-FU plus LV in two randomised studies (Carmichael *et al*, 2002; Douillard *et al*, 2002). In addition, UFT appears to have a more favourable toxicity profile than bolus 5-FU-based regimens and UFT has also been shown to be more acceptable than intravenous 5-FU in terms of patient preference (Borner *et al*, 2002). Both irinotecan and oxaliplatin have been used in combination with UFT in the treatment of patients with colorectal cancer, and have demonstrated promising activity and tolerability (Alonso *et al*, 2003; Bennouna *et al*, 2006).

A challenge facing physicians is that only about 50% of patients who receive first-line therapy are well enough to receive second-line chemotherapy (de Gramont *et al*, 2000; Douillard *et al*, 2000; Giacchetti *et al*, 2000; Saltz *et al*, 2000). One way forward, therefore, might be to combine a fluoropyrimidine with both oxaliplatin and irinotecan in the first-line setting so that a patient receives all three drugs ‘up front’. Previous studies of irinotecan and oxaliplatin with 5-FU have reported high response rates and impressive survival times (Falcone *et al*, 2002; Souglakos *et al*, 2006), suggesting that further investigation of concurrent administration of all three drugs is warranted in the first-line management of patients with metastatic colorectal cancer.

We undertook the present dose-finding study of UFT with LV plus alternating irinotecan and oxaliplatin with the aim of maintaining the efficacy demonstrated in previous 5-FU studies while improving the convenience and acceptability of the treatment by using UFT instead of intravenous 5-FU. We hypothesised that patients would benefit from receiving three of the most active drugs available for the treatment of this disease ‘up front’, as soon as they present with metastatic disease. By alternating the use of oxaliplatin and irinotecan, we hoped that patients would recover fully from the side effects of each drug before it was administered again. We also felt that it would be possible to increase the dose of oxaliplatin in an attempt to increase the efficacy of this regimen.

## MATERIALS AND METHODS

### Patient selection

Patients were eligible for inclusion in this single-centre, phase I open-label dose-finding trial if they had histologically confirmed advanced adenocarcinoma of the colon or rectum, with inoperable, measurable metastatic disease and no prior history of chemotherapy for metastatic disease other than adjuvant bolus 5-FU, which was permitted if administered more than 6 months before entry into this trial. Other selection criteria included age ⩾18 years, World Health Organisation performance status (PS) 0–2 and life expectancy >3 months. All patients were required to have adequate haematological function (neutrophil count ⩾1.5 × 10^9^ l^−1^ and platelet count ⩾150 × 10^9^ l^−1^), hepatobiliary function (serum bilirubin ⩽1.5 × upper limit of normal [ULN]; ALP ⩽5 × ULN; transaminase (AST or ALT) ⩽3 × ULN) and adequate renal function (estimated Cockcroft clearance ⩾50 ml min^−1^, or measured glomerular filtration rate [EDTA or creatinine clearance] in the normal range).

Patients were excluded if they had a concurrent uncontrolled medical illness or other previous or current malignant disease likely to interfere with protocol treatments. Patients could not have brain metastases, partial or complete bowel obstruction, chronic diarrhoea or inflammatory bowel disease, any confirmed abnormality of biliary transport, previous transplantation surgery or a recent history of uncontrolled angina or cardiac arrhythmias. Other excluded patient groups were pregnant or lactating women, patients on halogenated antiviral drugs or cytochrome P450 inhibitors and patients with a prior history of radiotherapy to the abdomen or pelvis.

The trial was conducted with full local ethical committee approval, according to the accepted standards of good clinical practice, and in agreement with the latest version of the Declaration of Helsinki. Written informed consent was a prerequisite for all patients. Pretreatment baseline evaluations included a complete medical history and physical examination (including weight and PS), full blood count and biochemistry including carcinoembryonic antigen (CEA) and radiological assessment with a computed tomographic scan.

### Treatment and dose escalation

Patients were assessed every 14 days when they attended for chemotherapy. Their PS and weight were noted and any toxicities recorded according to the National Cancer Institute Common Toxicity Criteria (NCI-CTC) version 2. A full blood count and biochemistry profile was also performed. The CEA assessment was repeated if the level was raised at baseline.

Treatment was initiated within 4 weeks of the investigations that were used for eligibility determination and disease evaluation. All patients received irinotecan 180 mg m^−2^ as a 90-min infusion on day 1 of the 28-day cycle, and were given UFT capsules (each containing tegafur 100 mg and uracil 224 mg) to take orally in combination with LV 90 mg day^−1^ three times daily on days 1–21, at a starting dose of 200 mg m^−2^ day^−1^. Patients returned on day 15 for a 2-h infusion of oxaliplatin at a starting dose of 85 mg m^−2^. Patients were recruited sequentially in cohorts of three and there was no intra-patient dose escalation. The planned dose escalation schedule is shown in Table 1.

If no grade 3 or 4 toxicity (other than alopecia) was encountered by the end of the first cycle, further patients were entered at the next dose level. If one or more patients developed grade 3/4 non-haematological toxicity, a further three patients were added to this cohort. Similarly, if one or more patients developed grade 4 haematological toxicity (other than anaemia), then a further three patients were added to this cohort. If there were no more episodes of grade 3/4 toxicity, then further patients were entered at the next dose level. When at least two out of six patients at the same dose level developed a significant toxicity, this was considered to be a dose-limiting toxicity (DLT) and the dose was the maximum administered dose; the level below this was therefore considered the maximum tolerated dose (MTD). If all patients in a cohort of three developed significant haematological or non-haematological toxicity, treatment was stopped and the dose level below was taken as the MTD.

Chemotherapy was administered for at least 8 weeks before reassessment of measurable metastatic disease by the Response Evaluation Criteria in Solid Tumours [RECIST] (Therasse *et al*, 2000), unless a criterion for study discontinuation was met. Patients with stable disease or a response continued treatment until there was clinical or radiological evidence of disease progression or the occurrence of unacceptable or cumulative toxicity. Treatment was stopped at the request of the patient for any reason or if, in the opinion of the investigator, it was in the patient's best interests to do so. Patients received 2 months of treatment initially and a further two periods of 2 months if the patient had stable disease or better on tumour assessment. Selected patients could receive treatment beyond a total of 6 months (until disease progression) at the discretion of the investigator and in agreement with the individual patient.

Prophylactic anti-emetics (dexamethasone 8 mg and ondansetron 8 mg) were administered intravenously with the irinotecan and oxaliplatin infusions and orally for 48 h afterwards. Delayed diarrhoea was treated early and aggressively with loperamide, with the addition of oral ciprofloxacin if it persisted for more than 24 h.

### Dose adjustments

On days 1 and 15, if ⩾grade 2 neutropaenia or platelets <100 × 10^9^ l^−1^ (< 75 × 10^9^ for oxaliplatin), all chemotherapy was delayed by 1 week. If >1 delay, or 1 delay of ⩾2 weeks occurred, the dose of irinotecan and oxaliplatin was reduced by 20% and UFT by one capsule (100 mg). This was continued at the lower dose for subsequent cycles unless further toxicity occurred. If a further delay for myelotoxicity occurred despite this dose reduction, a 50% reduction of the original dose of irinotecan and oxaliplatin was made if the patients' PS had not decreased. If this had occurred, withdrawal of the patient from this study was considered.

After an episode of severe diarrhoea (grade 3–4), chemotherapy was delayed until full recovery and resumed at irinotecan doses reduced by 20% and UFT reduced by one capsule. If diarrhoea from the previous cycle, even if not severe, had not resolved by the time the next cycle was due, treatment was delayed by 1 week. If further ⩾grade 3 diarrhoea occurred, irinotecan was reduced to 50% of the original dose and UFT reduced by two capsules. Grade 3 of above parasthesia of hands and feet and dysaesthesia in the throat, particularly in the cold, if persisting for 28 days (i.e., until the next cycle was due), led to omission of oxaliplatin from the regimen.

If hepatobiliary or renal function deteriorated below eligibility criteria limits during treatment, the irinotecan and/or oxaliplatin dose was adjusted as shown in Table 3. Any significant deterioration in liver function tests or renal glomerular filtration rate was promptly investigated by ultrasound examination to exclude progressive disease and also to look for possible reversibility of biliary tract or ureteric obstruction by stenting or percutaneous diversion.

## RESULTS

Twenty-five patients were entered into the study between February 2004 and April 2005. Patient demographics and clinical characteristics at baseline are shown in Table 2.

### Response

Twenty-one out of 25 patients were evaluable for response. Four out of 25 patients were not assessable for response. The first patient (dose level 1) died after only one dose of irinotecan and thus was not evaluable. Two patients received only one cycle of treatment before withdrawing from the study for psychosocial reasons; these patients were thus not assessable for response. One further patient (dose level 4) received only one dose of irinotecan and withdrew after being admitted to hospital with grade 3 abdominal pain.

Fifteen patients achieved a partial response (71%) and one patient underwent a partial hepatectomy. Two patients had stable disease and the clinical benefit to this cohort was 81% (complete response+partial response+stable disease). Four patients had progressive disease during treatment. Responses according to the individual dose levels are shown in Table 4. The median progression-free survival (PFS) was 268 days (8.8 months) (95% confidence interval 192–340 days (6.3–11.2 months)); overall survival data are too immature to be reported.

### Toxicity

Six patients were entered at dose level 1 and toxicity was assessed within the first month of treatment and used to dictate dose escalation (Table 5). The total number of cycles received at each dose level, together with dose reductions and delays, are detailed in Table 6. A total of 113 doses of irinotecan therapy were given to 25 patients. Thirty doses were reduced by 20% and one by 50%. Accordingly, 113 courses of UFT were prescribed, 27 of which were reduced by one capsule and one of which was reduced by two capsules as per protocol. A total of 94 doses of oxaliplatin chemotherapy were administered, of which seven doses were reduced by 20%. The dose intensity for this regimen is shown in Table 7.

The first patient entered into the study was a young woman with poor performance status (PS 2) and multiple sites of disease. This patient developed grade 3 diarrhoea, nausea and lethargy soon after receiving the first cycle of treatment and was admitted to hospital. She developed grade 4 neutropaenia and died suddenly. There were no subsequent occurrences of grade 3 or 4 toxicities within the first month of therapy. One patient at dose level 1 died suddenly in the third month of treatment as a result of a cardiac abnormality. He had no prior cardiac history and no symptoms of angina during the chemotherapy. This death was considered to be either incidental or due to fluoropyrimidine-induced vascular spasm. The second cohort of patients was treated with the same dose of irinotecan and oxaliplatin but with an increased dose of UFT (250 mg m^−2^). One patient in this group experienced grade 3 diarrhoea; a total of six patients were thus entered at this dose level. Four patients were then entered at dose level 3 and received an increased dose of oxaliplatin (100 mg m^−2^). One of these patients complied poorly and received only one cycle of treatment, necessitating their replacement with a fourth patient. No grade 3/4 toxicities were evident at this dose level.

The first patient treated at dose level 4 (UFT 300 mg m^−2^) developed multiple grade 3 toxicities on completion of the first cycle of treatment. A total of six patients were scheduled for entry at dose level 4, but each of the first four patients treated at this dose level suffered multiple grade 2 toxicities and a further two patients experienced a grade 3 toxicity. Further recruitment at this dose level was therefore stopped. The DLTs were lethargy, diarrhoea and vomiting. Dose level 3, that is irinotecan 180 mg m^−2^, oxaliplatin 100 mg m^−2^and UFT 250 mg m^−2^, was considered to be optimal and this dose level was expanded with a further five patients (nine patients in total). There were no grade 3 or 4 toxicities resulting from the first cycle of treatment at this dose level; however, two patients developed grade 3 toxicity with prolonged administration of chemotherapy (see Table 5).

With regard to neurotoxicity, 15 out of 25 patients developed grade 1 toxicity during therapy and one patient (dose level 3) experienced grade 3 toxicity during cycle 2, which necessitated a 20% reduction in the oxaliplatin dose for all subsequent cycles. There was no evidence of grade 2 or higher hand–foot syndrome or stomatitis.

## DISCUSSION

This phase I study has shown that the combination of UFT, irinotecan and oxaliplatin is a well-tolerated and efficacious first-line treatment for patients with metastatic colorectal cancer. Thus far we have shown an objective response rate of 71% and disease stabilisation in a further 10% of patients. One patient underwent a partial hepatectomy. As expected, the DLTs were lethargy, nausea and diarrhoea. There were no cases of hand–foot syndrome at any dose level, which underlines an advantage of UFT over continuous 5-FU regimens or capecitabine.

The response rate observed in our study was higher than that reported in a previous study of the combination of UFT, irinotecan and oxaliplatin in the first-line setting. In the study by Petrioli *et al* (2004), a fixed dose of UFT (250 mg m^−2^ day^−1^) was given for 28 days of a 35-day cycle, with oxaliplatin (85 mg m^−2^) on days 1 and 15 of odd numbered cycles and irinotecan (180 mg m^−2^) on days 1 and 15 of even numbered cycles. An objective response rate of 58.5% was achieved using this regimen and diarrhoea was the most common grade 3 toxicity, occurring in 29% of patients. We employed a higher dose intensity for both irinotecan and oxaliplatin, and achieved an improved response rate with an equally acceptable level of toxicity. The alternating use of oxaliplatin and irinotecan was deliberately chosen in our study to allow patients to recover fully from the side effects of one agent before it was administered again. Using this approach, we were able to increase the dose of oxaliplatin to 100 mg m^−2^ in an attempt to increase the efficacy of this regimen within the limits of tolerability. Dose-limiting neurotoxicity, including hand–foot syndrome, was not encountered during dose escalation and only one patient had grade 3 neurotoxicity at the MTD that required a dose reduction during the second treatment cycle.

Previous studies have examined the efficacy of concurrent treatment with irinotecan and oxaliplatin with 5-FU rather than UFT. Souglakos *et al* (2006) reported a 57% response rate when irinotecan and oxaliplatin were given ‘up front’ with 5-FU to a cohort of 57 patients. Falcone *et al* (2002) reported a higher response rate (71%) using a similar combination, as well as a 26.5-month overall survival and a 10.4-month PFS, which was comparable with the 8.8 months observed in our study. To our knowledge, there are no published studies combining irinotecan and oxaliplatin concurrently with the other major oral fluoropyrimidine, capecitabine, as a first-line treatment. There have been, however, a number of other phase I and II trials presented in abstract form using this triplet combination as a 3-week schedule, but the doses of each of the drugs varies widely between trials. Maroun *et al* (2006) reported early findings of a dose-escalation study for first-line treatment of metastatic colorectal cancer starting at irinotecan 180 mg m^−2^ day 1, oxaliplatin 85 mg m^−2^ day 1 and capecitabine 850 mg m^−2^ bid days 2–15, and discovered febrile neutropaenia to be the main DLT, but the MTD had not been reached.

While it is important not to over-interpret results from these studies, the response rates and median survival times suggest that further investigation of this ‘up front’ approach is warranted in patients with metastatic colorectal cancer, many of whom will not have the opportunity to receive a second-line treatment. While some patients may not be offered further treatment after the failure of first-line therapy and others may refuse it, the majority will have deteriorated clinically with disease progression during the first-line therapy to such an extent that they are unfit for further chemotherapy. Administering all three drugs ‘up front’ to patients with advanced colorectal cancer therefore maximises their chances of benefiting from three of the most active agents available for the treatment of this disease.

One possible criticism of this approach to treatment may be that patients who receive all three drugs in the first-line setting have limited possibilities for second-line therapy. On the other hand, it can be argued that this approach allows the rapid identification of patients with chemoresistant disease without the need for multiple levels of therapy. In addition, several options remain for second-line therapy, including rechallenge with concurrent oxaliplatin, irinotecan and UFT for patients who previously responded, treatment with the antiepidermal growth factor antibody cetuximab plus irinotecan (Cunningham *et al*, 2004) treatment with a combination of Mitomycin C and capecitabine or FOLFOX and bevacizumab (Giantonio *et al*, 2005) if parasthesia is not significant.

In conclusion, we have established from this phase I study an MTD of irinotecan 180 mg m^−2^ on day 1, oxaliplatin 100 mg m^−2^ on day 15 and UFT 250 mg m^−2^ day^−1^ with LV 90 mg day^−1^ on days 1–21 of a 28-day cycle for the first-line treatment of metastatic colorectal cancer. This combination, which provides a high response rate, prolonged PFS and a good side-effect profile, is being investigated as part of an ongoing phase II trial.

## Figures and Tables

**Table 1:** Dose^a^ escalation cohorts

**Cohort**	**UFT (mg m^−2^ day^−1^)**	**Irinotecan (mg m^−2^ day^−1^)**	**Oxaliplatin (mg m^−2^ day^−1^)**
1	200	180	85
2	250	180	85
3	250	180	100
4	300	180	100

a Doses were calculated using the patient's body surface area with no upper limit. UFT was administered on days 1–21 of the 28-day cycle; irinotecan was administered on day 1 and oxaliplatin was administered on day 15.

**Table 2:** Patient characteristics at baseline

No. of patients	25
Median age, years (range)	63 (24–79)
Sex, no. of male/female subjects	18/7
	
*Primary tumour site, no. of patients*	
Colon	13
Rectum	6
Rectosigmoid	6
	
*WHO performance status, no. of patients*	
0	7
1	16
2	2
	
*Prior surgical resection, no. of patients*	
Radical	8
Palliative	5
Defunctioned only	1
None	11
	
*Sites of metastatic disease, no. of patients*	
Liver only	10
LN only	1
Liver and lung	3
Liver and LN	1
Lung and LN	2
Liver, lung and LN	4
Pelvic disease only	2
Pelvic and liver	1
Pelvic and LN	1
	
*No. of measurable lesions (no. of patients)*	
1	3
2	1
⩾3	21

LN=lymph node; WHO=World Health Organisation.

**Table 3:** Dose adjustments

**Day 1 and 15 lab values**		**Irinotecan (%)**	**Oxaliplatin (%)**	**Uft (%)**
*Bilirubin* a	*ALP* a			
<1.5 × *n*	<5 × *n*	100	100	100
1.5–3 × *n*	>5 × *n*	50	100	Omit
>3 × *n*	—	Omit	Omit	Omit
				
*Cockcroft (GFR; ml min^−1^)* b				
>50		100	100	100
30–50		100	50	100
<30		50	Omit	1 cap reduction^c^

a Deteriorating liver function during treatment could indicate progressive disease or biliary obstruction and was investigated by ultrasound examination.

b Deteriorating renal function during treatment could indicate progressive pelvic disease and ureteric obstruction and was investigated by ultrasound examination.

c UFT is available in 100 mg capsules. All calculated doses were rounded to the nearest no. of capsules.

**Table 4:** Antitumour efficacy

	**Dose level 1**	**Dose level 2**	**Dose level 3**	**Dose level 4**	**Total**
**Response**	**(*n*=6)**	**(*n*=6)**	**(*n*=9)**	**(*n*=4)**	**(*n*=25)**
Complete response (CR)	0	0	0	0	0
Partial response (PR)	2	4	7	2	15
Stable disease (SD)	1	0	1	0	2
Progressive disease	2	1	0	1	4
Not assessable	1	1	1	1	4
					
Objective response rate^a^	71%				
Clinical benefit (CR+PR+SD)^a^	81%				

a 21 evaluable patients.

**Table 5:** Worst toxicity per patient (all cycles) by dose level

**Dose level**	**1 (*n*=6)**			**2 (*n*=6)**			**3 (*n*=9)**			**4 (*n*=4)**		
**NCI-CTC grade**	**2**	**3**	**4**	**2**	**3**	**4**	**2**	**3**	**4**	**2**	**3**	**4**
Anaemia	1			1			2			1		
Leucopaenia			1							2		
Neutropaenia			1	1						1		
Thrombocytopaenia		1										
Alopecia											1	
Anorexia	1											
Lethargy	2	1		2			4			2	1	
Nausea	1	1		1			1				1	
Vomiting	1	1		1			1				1	
Diarrhoea	2	2		3	1			1		1	3	
AST	2											
Neuropathy								1				
Infection			1									
Abdominal pain		1									1	
Cardiac			1									

AST=aspartate aminotransferase; NCI-CTC=National Cancer Institute Common Toxicity Criteria.

**Table 6:** Number of cycles given, number of dose reductions and delays

		**Irinotecan cycles**				**Oxaliplatin cycles**				**UFT cycles**				
**Dose level**	**No. of patients**	**Total**	**Median (range)**	**20% DR**	**50% DR**	**Total**	**Median (range)**	**20% DR**	**50% DR**	**Total**	**Median (range)**	**1 capsule DR^a^**	**2 capsules DR^a^**	**Delays Weeks**
1	6	22	3 (1–6)	4	0	17	2 (0–6)	0	0	22	3 (1–6)	3	0	3
2	6	27	6 (1–6)	13	0	23	4 (1–6)	2	0	27	6 (1–6)	10	0	5
3	9	46	6 (1–6)	2	0	43	6 (0–6)	3	0	46	6 (1–6)	3	0	4
4	4	18	5.5 (1–6)	11	1	11	3 (0–5)	2	0	18	5.5 (1–6)	11	1	3
Total	25	113	—	30	1	94	—	7	0	113	—	27	1	15

a Each UFT capsule contained tegafur 100 mg and uracil 224 mg.

DR: dose reduction.

**Table 7:** Median dose intensity (%) over two, four and six cycles

	**Two cycles**		**Four cycles**		**Six cycles**	
**Dose level**	**Irinotecan**	**Oxaliplatin**	**Irinotecan**	**Oxaliplatin**	**Irinotecan**	**Oxaliplatin**
1	100	98.2	89.6	92.1	89.5	94.5
2	100	100	82.8	82	81.8	92.3
3	100	100	100	100	100	100
4	80	66.7	85	56	85.9	62.6
